# Ferroptosis Biomarkers in HPV-Negative Head and Neck Squamous Cell Carcinoma

**DOI:** 10.3390/cancers18142320

**Published:** 2026-07-18

**Authors:** Sarah M. Parks, Werner J. Geldenhuys, Scott A. Weed

**Affiliations:** 1 Department of Biochemistry and Molecular Medicine, School of Medicine, West Virginia University, Morgantown, WV 26506, USA; sarah.spear@hsc.wvu.edu; 2 Department of Pharmaceutical Sciences, School of Pharmacy, West Virginia University, Morgantown, WV 26506, USA; 3 Graduate Program in Cancer Cell Biology, West Virginia University, Morgantown, WV 26506, USA

**Keywords:** HNSCC, HPV-negative, ferroptosis, biomarker

## Abstract

**Background/Objectives**: Head and neck squamous cell carcinoma (HNSCC) is an aggressive cancer of the oral cavity, pharynx, larynx and upper airway. HNSCC is subdivided into distinct clinical entities based on the presence or absence of high-risk human papillomavirus (HPV) infection. While HPV-positive HNSCC patients respond better to conventional and targeted therapies than HPV-negative cases, recurrence is more frequent in HPV-negative cases with limited treatment options. Ferroptosis is a form of iron-based programmed cell death caused by excessive lipid peroxide accumulation. Biomarkers of ferroptosis have proven useful for identifying and triggering ferroptosis in models of treatment-resistant cancers. Identifying and understanding ferroptosis biomarker function in HPV-negative HNSCC allows the possibility for more effective treatment strategies. **Methods**: Literature searches combined with the public online ferroptosis database FerrDB V3 were utilized for the objective identification and classification of established and non-established ferroptosis biomarkers in HPV-negative HNSCC. Additional novel biomarkers were identified based on known roles in HNSCC oncogenesis. **Results**: A total of 30 ferroptosis biomarkers were identified associated with HPV-negative disease. Biomarkers were grouped according to shared biological functions, with each marker summarized for prognostic and mechanistic roles in HNSCC ferroptosis. **Conclusions**: The described biomarkers present a means for stratifying tumors for ferroptotic induction, with several offering potential novel methods for overcoming refractory HPV-negative disease. These biomarkers may warrant further investigation into devising future patient-tolerant strategies that selectively activate ferroptosis in HPV-negative HNSCC.

## Simple Summary

Human papillomavirus (HPV)-negative head and neck squamous cell carcinoma (HNSCC) is an aggressive cancer resistant to conventional and current targeted therapies. Ferroptosis is an iron-based form of cell death that is typically suppressed in therapy-resistant cancers. This review summarizes biomarkers selected with the aid of the ferroptosis database FerrDB V3 for roles in HPV-negative HNSCC as possible tissue biomarkers to aid in diagnosis or drug treatment stratification. It also aims to evaluate the impact of the different contributing proteins in ferroptosis on clinical outcomes and the pharmacological landscape. The identified biomarkers may be of diagnostic and prognostic value in identifying HPV-negative HNSCC with increased disease resistance, as well as providing rational candidates for the development of novel anti-cancer chemotherapeutics targeting ferroptosis.

## 1. Introduction

### 1.1. Head and Neck Squamous Cell Carcinoma

Head and neck squamous cell carcinoma (HNSCC) collectively encompasses tumors of the upper aerodigestive tract originating from the lining squamous epithelium. HNSCC is the most common cancer type impacting the head and neck region, accounting for 900,000 new cases annually worldwide with approximately 450,000 deaths [1]. Primary risk factors for HNSCC include tobacco smoke and smokeless tobacco use, alcohol consumption, betel quid/areca nut chewing, types of salted fish, airborne pollutants and infection with high-risk human papillomavirus (HPV) [2,3,4,5,6]. Viral status is used to clinically stratify HNSCC into HPV-positive oropharyngeal and HPV-negative disease, with each subtype having its own treatment guidelines [7]. The etiology of HPV-positive HNSCC is due to the inactivation of the retinoblastoma (Rb) and tumor protein 53 (TP53), tumor suppressors by the E7 and E6 viral oncogenes, respectively, leading to genomic instability that inactivates cellular tumor suppressors and activates oncogenic drivers [8]. HPV-negative disease is primarily due to mutational accumulation caused by a variety of environmental DNA damaging agents, with the tobacco-associated carcinogens acrylonitrile, nitrosamines and polycyclic aromatic hydrocarbons most common [9]. Genomic evidence further indicates that HPV-negative HNSCC has a larger mutational load with a higher degree of copy number variants (CNVs) that impact tumor suppressive and oncogenic genes [10]. Standard-of-care for all HNSCC includes surgery, chemotherapy and radiation, with HPV-positive cases responding more favorably with better long-term survival than HPV-negative patients [4]. The more widespread mutational profile in HPV-negative disease largely underlies treatment resistance, where such patients typically experience more frequent and shorter times to disease recurrence [11]. To date, only two classes of targeted therapeutics are approved by the United States Food and Drug Administration (FDA) for locally advanced, recurrent or metastatic disease: Cetuximab, which targets the epidermal growth factor receptor (EGFR) and the anti-programed death-1 (PD-1) immune checkpoint inhibitors nivolumab and pembrolizumab [12,13,14]. These monoclonal antibody inhibitors give mixed responses, with many patients displaying disease remission while others suffer from adverse events and/or lack of efficacy [15]. While additional omics-based work combined with informatic profiling has refined stratification of each clinical subtype into several specific molecular groupings with therapeutic potential [16,17], this information has yet to translate into new clinical treatment options. The paucity of targeted treatments for HNSCC, combined with acquired resistance to existing therapies, represents a persistent clinical barrier to improving patient outcomes.

### 1.2. Ferroptosis

Ferroptosis is a form of cell death initiated by free iron which is susceptible to Fenton chemistry [18]. The reactive oxygen species generated from free iron (ferrous; Fe^2+^) leads to lipid peroxidation and cell death [19]. Several cancer types accumulate high levels of iron and reactive oxygen species (ROS) to maintain accelerated growth as a byproduct of oncogenic metabolism [20]. Redox biology is a critical aspect of maintaining the balance between cell proliferation and quiescence [21]. While key to these biological functions, imbalances in redox levels leading to excessive ROS production is a potent mediator of several cell death programs [22]. A novel form of redox-based programmed cell death was identified in 2012 termed ferroptosis [23]. Distinct from apoptosis, ferroptosis is defined by three hallmarks: oxidation of polyunsaturated membrane phospholipids, the availability of redox-active iron, and the loss of lipid hydroperoxide repair capacity [20]. Rapid tumor growth frequently outstrips the ability of tumors to be sufficiently vascularized, leading to hypoxic conditions that generate excessive ROS, forcing tumors to develop antioxidant strategies [24]. Additionally, in HNSCC and other cancers, radiation treatment leads to increased ROS production in tumor cells, where resistant cells develop mechanisms to promote survival that ultimately leads to recurrence [25]. Several studies now indicate that ferroptosis plays such a protective role in multiple cancers, including HNSCC, where molecular components that confer ferroptosis resistance are frequently upregulated to combat the effects of excessive oxidation [,^26^,^27^]. This is supported by recent work in HNSCC, where inducing ferroptosis enhances radiotherapy [28]. In addition to protein drivers, ferroptosis is also controlled by epigenetic, transcription and post-transcriptional modifications [29,30]. Molecular regulators of ferroptosis can also govern other forms of cell death, inhibiting apoptosis, necrosis or pyroptosis under certain conditions [30]. Ferroptosis is also tied to immune system regulation, where modulation of ferroptotic death in immune suppressive cell types has been considered as a way to enhance cancer immunotherapy [30,31]. Mitochondria play an important role in ferroptosis, serving as a significant site for ROS generation as a byproduct of ATP production [20,32,33]. Mitochondrial-generated ROS reacts with free iron, resulting in the generation of free radicals that oxidize free fatty acids and other unsaturated lipids to drive lipid peroxidation pathways [34]. As such, understanding how mitochondria regulate ferroptosis and the identification of associated pathway biomarkers opens the door to potential new methods of cancer treatment, as selectively enhancing ferroptosis in tumor cells would lead to tumor cell death and a reduction in overall tumor burden [35]. To this end, several small molecule compounds have been identified that selectively induce ferroptosis in in vitro cancer cell systems, allowing evaluation in cancer biology to determine the molecular underpinnings of ROS-mediated resistance to cell death in hypoxic conditions [36].

### 1.3. Utility of Ferroptosis Biomarkers in HNSCC

Cancer biomarkers are biological molecules found within body fluids or tissues that can be objectively measured to identify the presence and status of neoplastic tissue [37]. These biomarkers have become essential aides in guiding more precise and improved treatment options that have positively impacted patient mortality and quality of life [38]. In addition to eliminating cells damaged by oxidation, ferroptosis is a potential treatment option for tumor therapy by utilizing biomarkers to identify and promote sensitivity to ferroptosis in cancer cells, or for determining how different ferroptosis biomarkers influence cancer progression and resistance [39]. Biomarkers for susceptibility or resistance to ferroptosis have been evaluated for treatment and prognostic value in other cancers including lung, breast, hepatocellular, and renal cell carcinoma [40]. Knowing the actionable effects and mechanistic role of tumor biomarkers is essential in developing individualized cancer treatment, as biomarkers can aid in determining an optimal treatment course as well as exposing avenues for new treatment options in more resistant cancer types [41]. Targeting resistant tumors to selectively trigger ferroptosis as a potential treatment option is an attractive premise, as such cells are typically resistant to apoptotic programs [42]. Work in cancer cell lines and animal models has demonstrated the feasibility of ferroptosis targeting, where small molecules such as erastin (inhibiting the cystine-glutamate transporter SLC7A11) and RSL3 (inhibiting the antioxidant enzyme GPX4) kill tumor cells resistant to standard-of-care treatments [43]. While these and other proteins have been well evaluated as promising ferroptosis targets in HNSCC [44], new targets are constantly emerging that further our molecular understanding while providing additional prognostic value.

The potential therapeutic importance and molecular mechanisms of ferroptosis in head and neck cancer overall has been previously reviewed in detail [45,46,47,48,49]. The focus of this review is on biomarkers with known or potential clinical value in HPV-negative disease, since HPV-positive HNSCC contains decreased GSH levels that render such cells more vulnerable to lipid peroxidation and ferroptosis sensitivity [50,51]. Additionally, HPV-negative HNSCC tumors are more hypoxic, with adaptations to the metabolic consequences of low oxygen availability necessary for cell survival [52]. These factors likely contribute to the treatment-refractory nature of HPV-negative HNSCC, where new therapeutic approaches are urgently needed to combat this most deadly form of the disease.

## 2. Methodology

### Selection of HPV-Negative HNSCC Biomarkers

**Figure 1:**
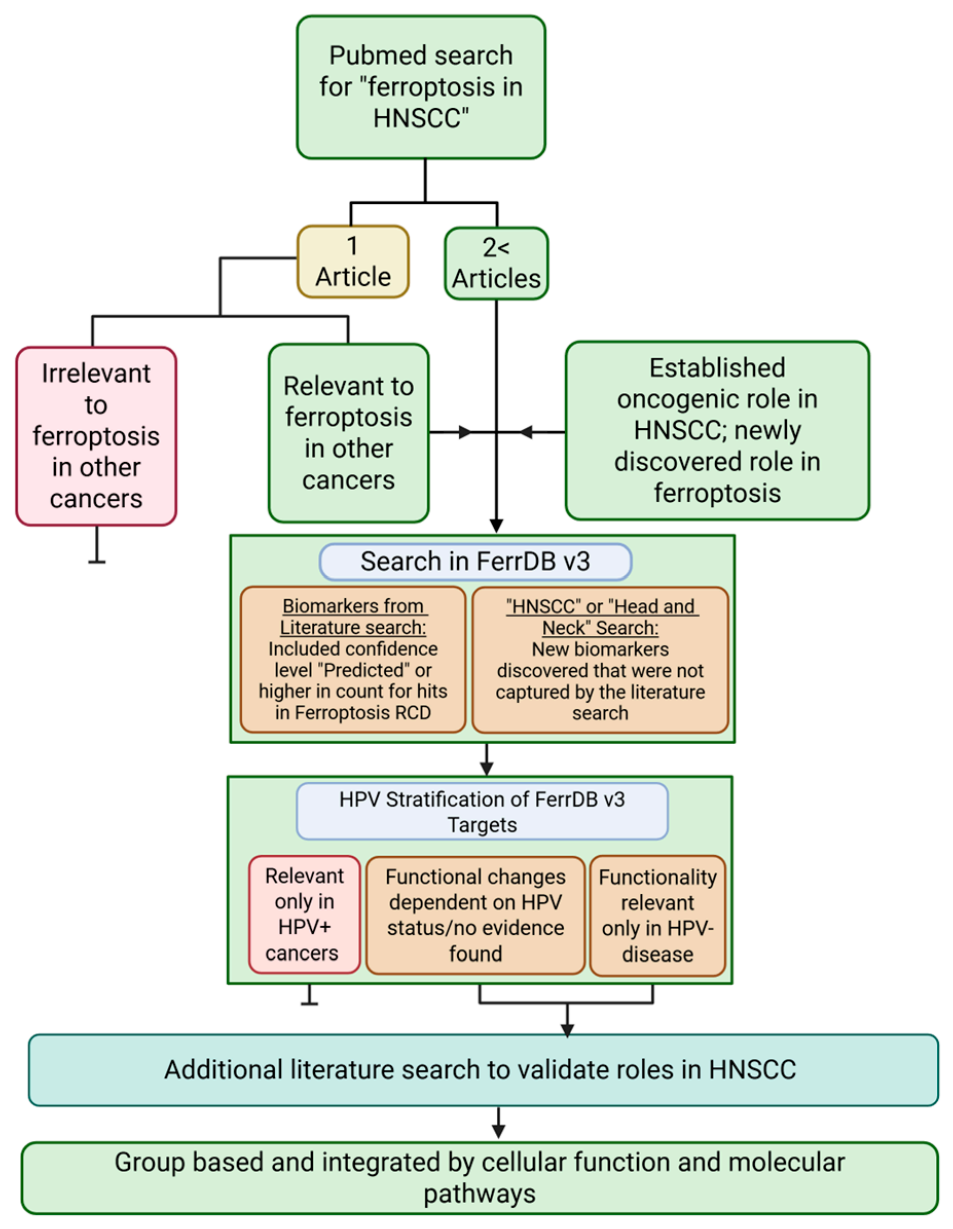
Selection of HPV-negative HNSCC Ferroptosis Biomarkers.

Computational-based selection of candidate biomarkers allows an unbiased approach in selecting clinically relevant candidates. Advances in combining imaging, informatic and computational approaches in HNSCC with machine learning are increasingly utilized for detection, prognosis and margin classification [53,54]. To this end, we utilized the FerroDb V3 database [55] to aid in identifying scientifically and clinically relevant proteins as ferroptosis biomarkers (Figure 1). To select an initial biomarker list for use in FerroDb V3, literature searches were conducted in Pubmed and focused on “Ferroptosis and HNSCC”. After compiling a list of several key articles, potential biomarkers were evaluated. If the biomarker was described in two or more research articles, it was included for further screening. Biomarkers with a single citation were further evaluated in the literature. If the biomarker was found to be irrelevant in other cancers within the context of ferroptosis, it was removed from further consideration. Relevant single-citation biomarkers were included for subsequent analysis. In addition, potential biomarkers were included if they had an established oncogenic role in HNSCC and a newly discovered role in ferroptosis.

Each potential biomarker from the literature search was further evaluated in FerrDB V.3. [https://www.zhounan.org/ferrdb/v3/pages/index.html, accessed on 22 June 2026] to determine confidence levels relating to roles exclusive to ferroptosis in the Regulated Cell Death (RCD) database. FerroDb V3 provides five categories of confidence: 1. Validated (“The most reliable. It requires convincing evidence from strict tests such as pharmacological or genetic inhibition or activation test”). 2. Screened (“No strict validation. Only high-throughput data is available”). 3. Predicted (“No strict validation. In the source article, the authors drew their conclusions based on computational results or their knowledge”) and 4. Deduced (“No strict validation. We, the curators, inferred the gene’s function based on our understanding of the source article”), and 5. GO (“An entry from the GO database”) [https://www.zhounan.org/ferrdb/v3/pages/help.html#confidencelevel, accessed on 22 June 2026]. Biomarkers exclusive to the Deduced and GO categories were excluded from further consideration. Additional candidates were identified by an independent search in FerrDB V3 for “Head and Neck” and “HNSCC”, categorized by the database as Driver, Suppressor, Marker, or Unclassified. Some biomarkers had no hits in FerrDB but were included if they fit the previous literature search criteria as they may be novel and not be present in the database.

Following FerrDB identification and validation, candidate biomarkers were subsequently stratified based on HPV status. Biomarkers exclusive to HPV-positive cancer were removed. Thirty biomarkers were either exclusive to HPV-negative HNSCC, were HPV-dependent, or had no determined HPV-status. These were selected for review and were grouped based on common cellular functions or roles in the same or similar molecular pathways (Table 1).

## 3. Ferroptosis Biomarkers in HPV-Negative HNSCC

### 3.1. Iron Regulation

#### 3.1.1. FTH1

**Figure 2:**
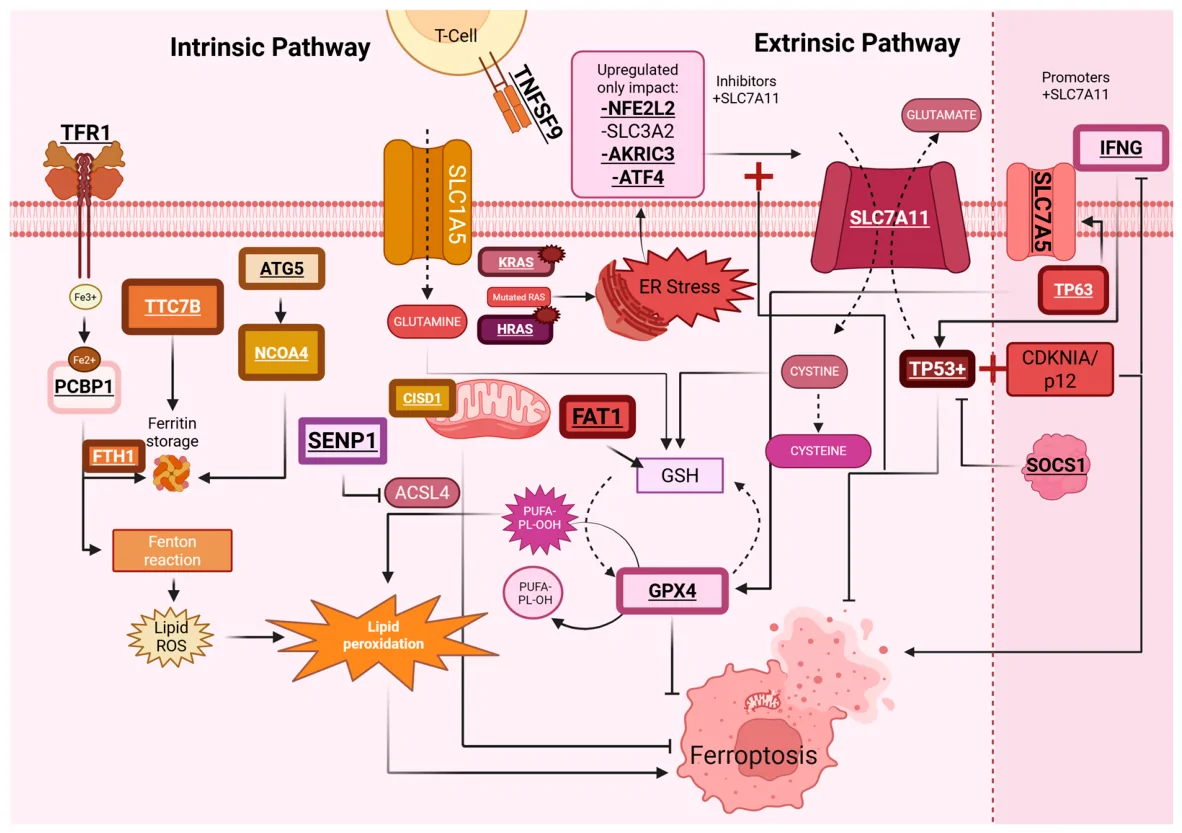
The intrinsic and extrinsic ferroptosis pathways. The intrinsic pathway involves the ferroptosis biomarkers TFR1, FTH1, TTCTB, ATG5, NCOA4, SLC1A5, SENP1 and CISD1. The extrinsic pathway includes KRAS, HRAS, NFE2L2, AKRIC3, ATF4, TP53, FAT1, GPX4, SLC7A11, SOCS1, SLC7A5, TP63 and IFNG. There is considerable overlap between the two pathways.

The ferritin heavy chain (FTH1) (Figure 2) is composed of 24 subunits with a mix of heavy and light chain subunits and is the major cytoplasmic intracellular storage component for soluble, nontoxic iron in cells [46,87]. FTH1 is important in iron homeostasis and for the prevention of oxidative damage through ferroxidase activity, where iron is oxidized from the ferrous [Fe^2+^] to ferric [Fe^3+^] form [87]. FTH1 is overexpressed in HNSCC compared to non-malignant epithelial cells and is associated with tumor progression and poor prognosis [88,89,90]. As such, FTH1 is a suppressor of ferroptosis in HNSCC that serves as an independent prognostic indicator [44,56,88]. Additional HNSCC neoplastic processes regulated by FTH1 included increased cell proliferation, tumor angiogenesis, cell migration, inflammation, extracellular matrix regulation, actin cytoskeletal regulation, and increased lymph node metastasis [88,91,92]. FTH1 serves as a significant prognostic marker for smoking-positive and alcohol use compared to smoking and alcohol-negative HNSCC [88]. Iron metabolism is key in governing ferroptosis, where iron storage by FTH1 has an inherently protective role that can be altered through FTH1 overexpression or by mutations in the FTH1 pathway that cause FTH1 upregulation [88,91]. The nuclear receptor coactivator 4 (NCOA4) mediates ferroptosis through binding to the ferritin complex, leading to lysosomal degradation [93]. This results in increased levels of free iron in the cytoplasm, leading to ferroptosis through upstream activation of ferritinophagy [30,46,93]. Silencing of iron-responsive element-binding protein 2 (IREB2) and the inhibition of metal regulatory transcription factor 1 (MTF1) through ataxia-telangiectasia mutated (ATM)-mediated phosphorylation promotes ferroptosis by reducing FTH1 transcription [30]. The BTB and CNC homology 1 (BACH1) transcriptional repressor is also able to promote ferroptosis through the inhibition of FTH1 transcript production [30]. Additionally, the tetratricopeptide repeat domain 7B (TTC7B) and protease-activated receptor 1 (PAR1) can upregulate expression of FTH1 through the JAK2/STAT3 pathway [83]. FTH1 overexpression levels have also been linked to a role in tumor immunity, where there is a positive correlation for infiltration with immune-suppressing M2 macrophage infiltration in solid tumors [44,56,89].

#### 3.1.2. TFR1

**Figure 3:**
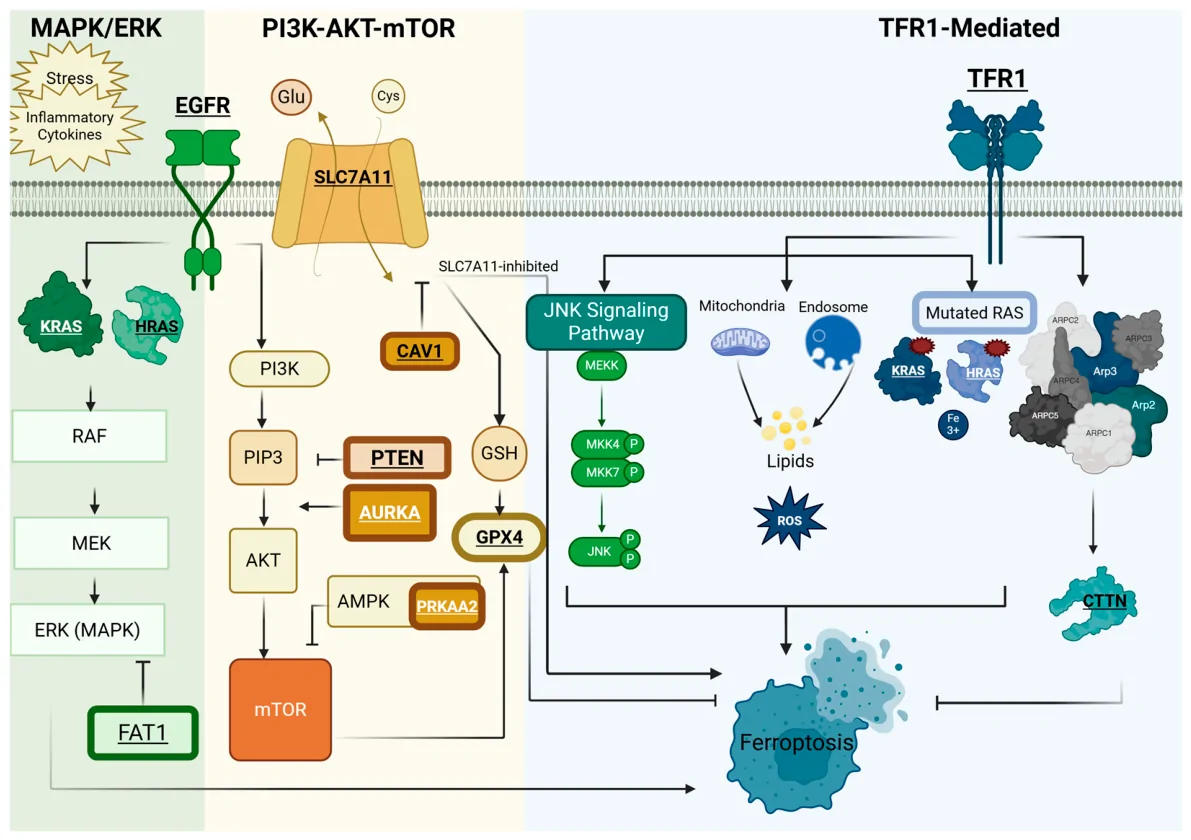
The MAPK/ERK, PI3K-AKT-mTOR, TFR1-mediated ferroptosis pathways. The biomarkers of the MAPK/ERK cascade include EGFR, KRAS, HRAS, and FAT1. The biomarkers of the PI3K-AKT-mTOR pathway include SLC7A11, EGFR, CAV1, PTEN, AURKA, GPX4 and PRKAA2. The biomarkers of the TFR1-mediated pathway include TFR1, KRAS, HRAS, and CTTN.

Transferrin receptor protein 1 (TFR1 or TFRC) (Figure 2 and Figure 3) is a transmembrane receptor responsible for cellular iron uptake, selectively binding and internalizing extracellular transferrin through clathrin-mediated endocytosis [94]. TFR1 is responsible for trafficking iron to the mitochondria, where it is utilized to create iron-sulfur clusters required for electron transport during oxidative respiration [20,95]. As a protein central to iron homeostasis, TRF1 is a critical regulator of ferroptosis. Increased iron uptake through TRF1 overexpression or increased endocytic recycling leads to iron overload that sensitizes cells to ferroptosis through increased Fenton reaction, enhancing ROS generation utilized in lipid peroxidation [96]. Tumor cells counteract the effect of increased iron uptake through rewiring iron metabolism to increase iron storage, enhancing antioxidation pathway activity or by altering lipid metabolism to reduce oxidative lipid damage [97]. TFR1 also interacts with the inhibitor of NF-κB (IKK) complex to enforce nuclear localization of NF-κB and subsequent oncogenic gene transcription [98]. In HNSCC, TRF1 overexpression is linked to worse prognosis, with recurrent patients having increased TFR1 levels [92]. Mutated HRAS and KRAS induce TFR1 endosomal cycling to increase internal iron levels necessary for ribonucleotide reductase function in DNA synthesis, but can further serve to enhance ferroptosis sensitivity [20,99]. While TFR1 is an attractive ferroptosis target for anti-cancer therapy, efficacy is currently limited due to concerns regarding anemia and increased T-cell apoptosis in instances where TRF1 in inhibited [100].

#### 3.1.3. ATG5

Autophagy-related 5 (ATG5) (Figure 2) is an E3 ubiquitin ligase and key autophagy regulator [101]. ATG5-mediated autophagy is responsible for ferritin degradation, where excessive iron release from elevated ATG5 activity leads to the Fenton reaction, causing oxidative damage and leading to ferroptotic cell death [20]. While ATG5 expression is associated with increased ferroptosis sensitivity in HPV-negative nasopharyngeal carcinoma, high ATG expression promotes increased HNSCC cell proliferation [58]. Accordingly, high ATG5 expression is associated with decreased overall survival and poor prognosis [46,58,59]. Additionally, ATG5 may be linked to expression of the mitochondrial ribosomal protein L (MRPL) family members MRPL13, 14 and 18, mitochondrial ribosomal proteins essential for mitochondrial protein translation. ATG5 is also an immune infiltration marker in HNSCC, where elevated ATG5 is positively correlated with PD-L1/PD-L2 signaling and decreased CD8+ T cells [58,102]. As a result, patients with high ATG5 levels may not benefit from immune checkpoint inhibitor (ICI) treatment due to a predicted decreased ICI response [58].

#### 3.1.4. NCOA4

**Figure 4:**
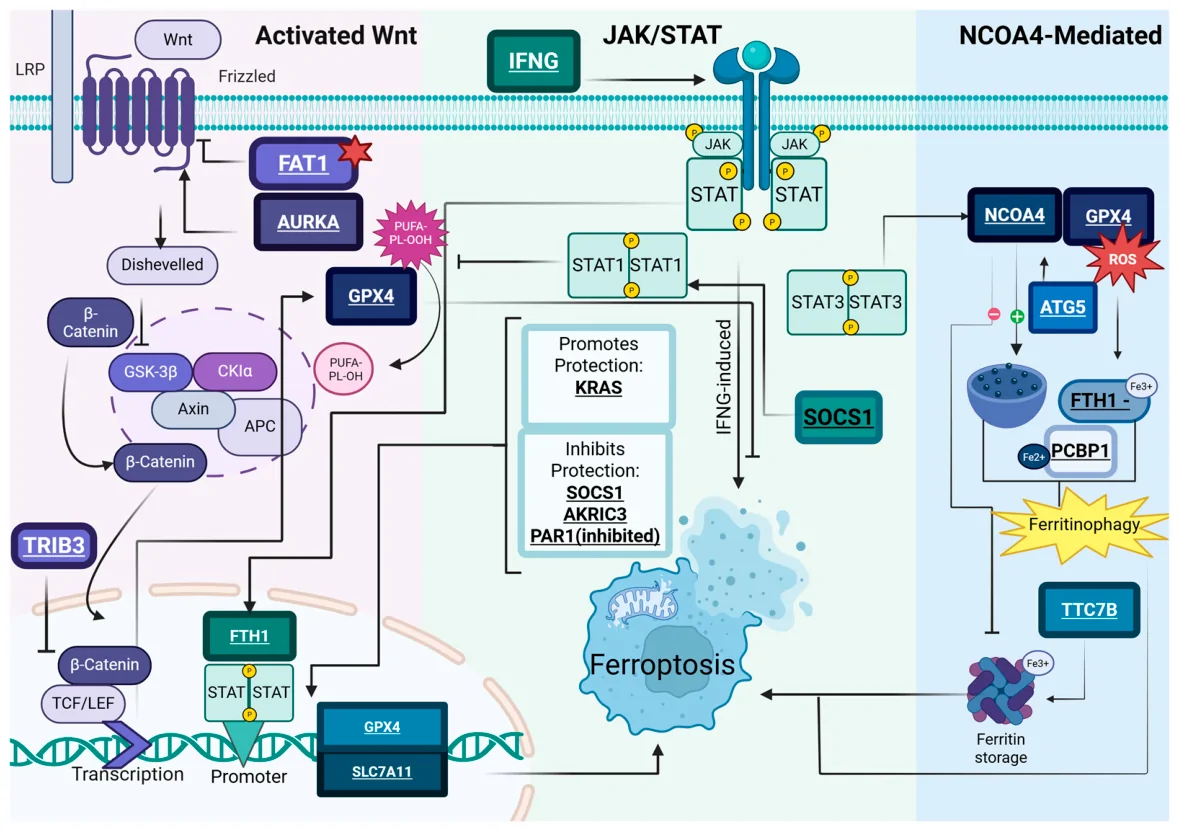
The Activated Wnt/β-Catenin, JAK/STAT, and NCOA4-mediated pathways of ferroptosis. The biomarkers of the activated Wnt/β-Catenin pathway include FAT1, AURKA, GPX4, TRIB3 and FTH1. The biomarkers of the JAK/STAT generalized pathway include IFNG, SOCS1, KRAS, AKRIC3, PAR1, GPX4, and SLC7A11. Biomarkers of the NCOA4-mediated pathway include NCOA4, GPX4, ATG5, PCBP1, FTH1, and TTC7B.

Nuclear receptor coactivator 4 (NCOA4) (Figure 4) is an intracellular receptor that binds FTH1 to mediate autophagic FTH1 destruction and release of cytoplasmic free iron by ferritinophagy [103]. Accordingly, NCOA4-dependent ferritin degradation or NCOA4 overexpression activates ferroptosis [20,29]. Ferroptosis is enhanced in HNSCC when NCOA4 is upregulated, resulting in increased ROS production and decreased FTH1 levels [104]. Furthermore, elevated NCOA4 expression has been identified as a prognostic marker in HNSCC for improved outcomes and decreased cell proliferation [20,103]. In agreement with this work, low NCOA4 has been identified as a gene signature component predictive of radioresistance in HPV-negative HNSCC patients and cell lines [57]. NCOA4 downregulation in HNSCC is mediated in part by the overexpression of oncogenic microRNA miR-182-5p, resulting in ferroptosis suppression and increased tumor cell survival [104].

#### 3.1.5. PCBP1

Poly(rC)-binding protein 1 (PCBP1) (Figure 4) is an RNA-binding protein that regulates gene expression through alternative splicing, transcript stability, and translational and transcriptional control [105,106]. PCBB1 also binds iron, severing as a chaperone to deliver iron to ferritin and other iron-binding storage proteins and enzymes [107]. The iron transport function of PCBP1, in conjunction with BolA2, is central to preventing oxidative damage by facilitating the formation of iron-sulfur clusters [108,109]. In cancer, PCBP1 acts as a tumor suppressor by stabilizing tumor suppressor transcripts and suppressing oncogenic gene expression [110,111,112,113]. In HPV-negative HNSCC cells and xenografts, PCBP1 prevents ferritinophagy-mediated ferroptosis by blocking poly unsaturated lipid peroxidation through binding the 3′-UTR regions of the ferroptotic-inducing BECB1 and ALOX15 transcripts to repress their translation [60]. The PCBP1 suppressive effect on ferroptosis was reversed by siRNA-mediated PCBP1 knockdown or with the anti-inflammatory drug sulfasalazine, suggesting a potential novel treatment method of ferroptosis re-sensitization in tumors.

### 3.2. Antiporters

#### 3.2.1. SLC7A11

Solute Carrier Family 7 Member 11 (SLC7A11) or xCT (Figure 2 and Figure 3), is the light chain subunit of the cystine-glutamate antiporter involved in the extracellular uptake of cystine utilized for glutathione synthesis to protect cells from oxidative stress [30,44,46,91,114]. The SLC7A11 gene is located on chromosome 4 in humans, with the protein having 12 transmembrane domains [114]. SLC7A11 is a key inducer of the antioxidant system of ferroptosis by inhibiting the extrinsic/transporter dependent pathway [30,44]. SLC7A11 is the light chain of the xCT system, where together with the heavy chain solute carrier family 3 member 2 (SLC3A2), it regulates the production of reduced glutathione (GSH) crucial in maintaining redox balance [20,30,114]. SLC7A11 expression is commonly elevated in cancer compared to normal tissues [114]. Various cellular stress-inducing conditions can impact SLC7A11 expression at the post-transcriptional and translational levels, including metabolic stress, amino acid starvation and hypoxia [114]. SLC7A11 transcription is regulated by nuclear factor erythroid 2-related factor 2 (NRF2) and activating transcription factor 4 (ATF4). Proteins that interact with SCL7A11 to modify transporter activity and stability include tumor protein 53 (TP53), suppressor of cytokine signaling 1 (SOCS1), BRCA1-associated protein 1 (BAP1), cluster of differentiation 44 (CD44), SLC3A2, AT-rich interaction domain 1A (ARID1A) and protein regulator of cytokinesis 1 (PRC1) [20,39,44,62,91,114]. SLC7A11 overexpression in HNSCC is associated with the presence of lymph node metastasis and decreased overall survival [114]. In breast, pancreatic, and head and neck cancers, SLC7A11 overexpression maintains redox homeostasis in hypoxic conditions [91,114,115]. Accordingly, increased SLC7A11 expression due to NRF2 upregulation in HNSCC generates a ferroptosis-resistant pathway that confers radiation resistance [39,46,116]. Interestingly, levels of SLC7A11 tend to be higher in HPV-negative than HPV-positive HNSCC patients, likely contributing to the lower radiation response typically seen in HPV-negative disease [91]. SLC7A11 can be transcriptionally activated by interleukin (IL)-6 through the JAK/STAT3 pathway to promote ferroptosis resistance and tumor development [44,76]. SLC7A11 can be inhibited by a variety of pharmacological compounds, including erastin, HG106, sulfasalazine, sorafenib and L-selenocysteines to trigger ferroptosis [30,91]. This has been demonstrated in HNSCC, where treatment resistance due to increased SLC7A11 expression has been overcome by sulfasalazine-induced ferroptosis [44,117].

#### 3.2.2. SLC7A5

Solute carrier family 7 member 5 (SLC7A5) or large amino acid transporter 1 (LAT1) (Figure 2) is a transporter for neutral essential amino acids [93]. SLC7A5 activity is important for cell growth, metabolism, and development as it is responsible for the transporting eight of the nine essential amnio acids through regions such as the blood–brain barrier and the placenta during human development [93]. In cancer, SLC7A5 is commonly overexpressed, especially in the context of the mammalian target of rapamycin (mTOR) activity where glutamine, arginine, and leucine are required for activation [93]. Overexpression of SLC7A5 increases tumor growth, correlating with increased tumor stages and worse outcomes in HNSCC, breast and gastric cancer patients [64]. SLC7A5 overexpression confers ferroptosis resistance by maintaining dynamic amino acid homeostasis to prevent oxidative stress [64,118]. SLC7A5 expression positively correlates with SLC7A11 overexpression, with SLC7A11 and tumor protein (TP)63-positivity reinforcing ferroptosis resistance [61,64]. Similar to SLC7A11, SLC7A5 binds the ferroptosis-inducing compound erastin at lower affinities, aiding in ferroptotic induction [39,64]. SLC7A5 upregulation also leads to increased resistance to immunotherapy, with TP63+/SLC7A5+ patients having increased infiltration of immune suppressive cell types in the TME [64].

### 3.3. Redox Regulation

#### 3.3.1. GPX4

Glutathione peroxidase 4 (GPX4) (Figure 3 and Figure 4) is responsible for maintaining redox homeostasis through antioxidant activity towards intracellular lipid reactive oxygen species (ROS) by conversion to lipid alcohols [20,39,116]. GPX4 acts in the cytoplasm where it is involved in mitochondria ROS regulation [44]. GPX4 is the canonical downstream intrinsic regulator of GSH to maintain oxidative homeostasis, preventing excessive ROS formation and subsequent lipid peroxidation [20,29,30,46,91]. GPX4 reduces phospholipid hydroperoxide production by functioning as a phospholipid hydroperoxidase, utilizing reduced GSH to reduce reactive lipid peroxides. [30,91]. Consequently, loss of GPX4 expression leads to ROS accumulation, which in turn can oxidize membrane lipids and lead to ferroptosis [20,46,61,114]. GPX4 is considered an oncogene, since expression levels are frequently elevated in HNSCC, as well as triple-negative breast, gastric and esophageal cancers, to produce ferroptosis resistance [20,119,120]. GPX4 upregulation in HNSCC cells that drives ferroptosis resistance also increases resistance to radiotherapy, resulting in poor prognosis while highlighting GPX4 as a potential therapeutic target [20,46,91]. HNSCC tumors with low GPX4 levels are associated with high immune checkpoint gene expression, increased ferroptosis and improved survival [77]. Conversely, several cancers downregulate GPX4 through glutaminolysis, metabolizing GSH through GPX4 when cysteine levels are decreased. This leads to increased iron concentration, elevated ROS levels and ferroptosis induction [102,121]. In this context, GPX4 is considered a tumor suppressor [122].

#### 3.3.2. CISD1

CDGSH iron-sulfur domain-containing protein 1 (CISD1) or mitoNEET (Figure 2), was identified through an off-target interaction of the type II diabetic drug pioglitazone [123,124]. CISD1 contains redox-active [2Fe-2S] clusters and is thought to function as a redox and pH sensor for the mitochondria [66,125]. Bioinformatic analysis shows that CISD1 is a ferroptosis-associated gene, where overexpression leads to tissue protection from ferroptosis [126]. CISD1 plays a role in mitochondrial redox homeostasis and bioenergetics. Knockdown or knockout studies show increased ROS production and a reduction in cell proliferation, whereas overexpression tends to increase cell proliferation [127,128]. Additionally, CISD1 is thought to play a role in mitochondrial turnover or mitophagy, where CISD1 is ubiquitinated on lysine residues through the PINK1/PARKIN process [129,130]. As CISD1 protects against ferroptosis and against mitochondrial lipid peroxidation, CISD1 inhibitors would be useful as adjuvant therapies to existing anti-cancer drugs by increasing ferroptosis sensitivity. CISD1 is necessary to prevent mitochondria-mediated ferroptosis by removing excess iron to prevent accumulation of both iron and mitochondrial lipid peroxides [20]. In addition, CISD1 inhibits ferroptosis through the mitochondria by limiting iron uptake [30]. Recently, CISD1 is an established prognostic biomarker in breast cancer, and high CISD1 expression in HNSCC corresponds with poor prognosis and increased immune evasion [131,132]. Additional studies in HNSCC have linked CISD1 expression to lymph node metastasis and gene amplification events associated with somatic mutations in TP53, IFNG, PTEN, and EGFR that supports tumor initiation, progression, growth and immune cell recruitment [131]. As such, anti-CISD1 approaches to induce ferroptosis may provide potential for more favorable response when paired with ICI therapies [131].

### 3.4. Transcriptional Regulation

#### 3.4.1. TP53

Tumor Protein 53 (TP53) (Figure 2) is a tumor suppressor gene that has diverse roles in protecting cells from the development and advancement of neoplastic insults [62]. The primary role of TP53 is to initiate G1 cell cycle arrest when DNA damage is present, permitting genomic errors to be corrected, or triggering apoptosis if damage cannot be corrected prior to passing the restriction point [62]. TP53 is the most frequently mutated gene in HNSCC, where mutations or deletions range from 39 to 67% of patients [62,133]. Most TP53 mutations are missense, where loss of function is linked to tumor development and resulting poor prognosis [59,61,62,133]. In colorectal cancer, non-small lung cancer, and glioblastoma, TP53 upregulation has been associated with ferroptosis resistance by impairing expression of SLC7A11 or GPX4, or through the promotion of anti-ferroptosis genes including the TP53 regulator mouse double minute 2 (MDM2) [39,62,134]. HPV-negative patients with TP53 mutations have dysregulation of multiple ferroptosis genes, serving to prevent ferroptosis and likely contributing to worse outcomes in patient groups [59,133]. HNSCC with elevated TP53 due to mutation is associated with immune cold tumors, exhibiting reduced macrophage, CD8+ and CD4+ T-cell infiltration [62]. TP53 is also able to mediate ferroptosis through different members of the arachidonate lipoxygenase (ALOX) family, utilizing ALOX12 for TP53-medidated downregulation of SLC7A11 that drive the extrinsic ferroptosis pathway [30]. TP53 can also induce ferroptosis through the inhibition of SLC7A11, which provides resistance to ferroptosis induced by Golgi stress [30,135]. Finally, TP53 can regulate lipid peroxidation through spermidine *N*^1^-acetyltransferase 1 (SAT1) or glutaminase 2 (GLS2) to promote ferroptosis [30,136].

#### 3.4.2. ATF4

Activated transcription factor 4 (ATF4) (Figure 2) is initiated when the integrated stress response is triggered by amino acid deprivation, viral infection or endoplasmic reticulum (ER) stress [67]. ATF4 has multiple retrocopies in the human genome that can produce functional transcripts in adaptation for tumor cell survival and maintenance of homeostasis, or serve as an inducer of apoptosis or ferroptosis when under persistent cellular stress [67]. ATF4 upregulation during ER stress generates a feedback loop that transcribes SLC7A11, inhibiting stress-produced ROS to protect against ferroptosis [29,30,39,114]. The response of ATF4 to cellular distress allows SLC7A11 to generate a response condition where cancer cells are able to survive under low glucose conditions [114]. However, this mechanism enhances the potential for cells to become resistant to ferroptosis when ATF4-SLC7A11 is upregulated in cancer cells [114]. In HNSCC, ATF4 works in conjunction with general control nonderepressible 2 (GCN2) to drive tumor cell proliferation, homeostasis, and tumor cell survival [67]. Inhibition of this pathway by the novel inhibitor AST-0513 in multiple HPV-negative HNSCC cell lines causes cells to undergo cell cycle arrest and subsequent apoptosis, presenting a potential treatment option [67].

#### 3.4.3. NFE2L2

Nuclear Factor, Erythroid 2 Like 2 (NFE2L2) (Figure 3) encodes the basic leucine zipper transcription factor NRF2 that is a master regulator of the antioxidant response [137]. Under homeostatic conditions, constitutively expressed NRF2 is bound to the oxidative stress sensor KEAP1 in the cytoplasm, where KEAP1-mediated NRF2 ubiquitination maintains low intrinsic levels [138,139]. Upon electrophilic or oxidative stress when autophagy is suppressed, KEAP1 binds to sequestosome 1 (SQSTM1; p62) that prevents KEAP1 from binding NRF2, permitting NRF2 to translocate to the nucleus to transcribe a variety of genes involved in ROS detoxification, iron and glutathione metabolism [140]. These include the ferroptosis genes FTH1, SLC7A11 and GPX4 among others, conferring ferroptosis resistance [141]. In cancer, mutations in NFE2L2 that prevent KEAP1 binding and suppress KEAP1 expression result in constitutive NRF2 nuclear localization, leading to elevated ferroptosis gene expression, giving rise to ferroptosis resistance [137,141]. Accordingly, hyperactivation of the NRF2 pathway is present in HNSCC, where it is associated with radioresistance in preclinical models of HPV-negative disease [142,143]. Treatment of cisplatin-resistant HPV-negative HNSCC cells with the anti-malarial drug artesunate induces ferroptosis to a moderate degree without effecting normal cells, with ferroptotic killing further enhanced when NRF2 is downregulated [68]. Such results support ongoing research into selective NRF2 targeting as an anti-cancer approach [144].

#### 3.4.4. TP63

Tumor protein 63 (TP63) (Figure 2) is a master regulatory transcription factor closely related to TP53, controlling chromatin regulation, apoptosis and cell cycle [145]. TP63 has six different isoforms, where the isoform lacking the amino terminus p53 homology domain (ΔNp63) is expressed in the basal layer of squamous epithelium to drive keratinocyte differentiation [146,147]. TP63 (ΔNp63) overexpression is considered oncogenic in squamous cell carcinomas [148,149], wherein HNSCC tissues and HPV-negative cell lines with TP63 overexpression are associated with increased proliferation, tumor cell survival and poor outcomes [150,151,152]. Subsequent findings in transgenic mice with TP63 knockout and carcinogen-induced tumors suggests that TP63 serves a tumor suppressive role, where loss of TP63 expression drives HNSCC progression and metastasis through upregulation of MAPK and STAT3 activity [153]. While the oncogenic and tumor suppressive roles of TP63 in HNSCC remain to be fully clarified, comprehensive evaluation of HNSCC gene expression from single-cell RNA sequencing in Gene Expression Omnibus (GEO) and transcript expression in the TCGA database indicated that TP63 and SLC7A5 were upregulated in patient tumors following immunotherapy [64]. TP63 overexpression in HPV-negative cell lines suppressed cellular iron levels and ferroptotic cell death. These laboratory-validated computational data suggest that TP63 serves as a transcription factor to upregulate SLC7A5 as a mechanism of ferroptosis resistance in HPV-negative HNSCC patients, consistent with roles for TP63 in upregulating GPX4 and ALDH3A1 to evade ferroptosis in other cancer types [154,155,156].

### 3.5. Cellular Metabolism

#### 3.5.1. AKR1C3

Aldo-keto reductase family 1 member C3 (AKR1C3) (Figure 2) is a member of the aldo-keto reductase family superfamily that utilizes NDAH or NADPH to catalyze aldehydes and ketones to their alcohol forms [157]. AKR1C3 specifically converts estrone and PGD2 into testosterones and estrogens, which in turn drive the progression of hormone-dependent cancers [158]. AKR1C3 also drives non-hormone dependent cancers through stimulating NFkB, MAPK and Akt signaling [158]. Elevated AKR1C3 levels in hepatocellular carcinoma prevent ferroptosis by increasing YAP nuclear translocation to upregulate SLC7A11 [159]. In addition, AKR1C3 binds heat shock protein family A member 5 (HSPA5) in the nucleotide binding domain, causing a chain reaction that leads to the stabilization of GPX4 and GSH production and ferroptosis inhibition [70]. The GPX4/HSPA5 axis is stabilized by AKR1C3, which in turn prevents formation of reactive oxygen species (ROS) and lipid peroxidation [30]. ARK1C3 is frequently overexpressed in HPV-negative HNSCC, correlating with poorer overall survival [71]. AKR1C3 overexpression contributes to radioresistance, where increased ROS production is attenuated by increased STAT pathway activity that reduces apoptotic and ferroptotic cell death [70]. Selective ARK1C3 inhibition in HNSCC enhances cell killing by cisplatin, suggesting a novel means to overcome chemotherapy and potentially radiation resistance [71].

#### 3.5.2. PRKAA2

Protein kinase AMP-activated catalytic subunit alpha 2 (PRKAA2) (Figure 3) is the catalytic subunit of AMP-activated protein kinase (AMPK), functioning as an energy consumption sensor and monitor of energy status by maintaining a homeostatic AMP/ATP ratio [160]. In conditions of low ATP, PRKAA2 is phosphorylated by the tumor suppressor LKB1 to stimulate ATP production [161]. AMPK activity in turn governs cell growth and proliferation while inhibiting mTOR activity [160,162]. Increased PRKAA2 expression influences cancer in a context-dependent manner, serving as a tumor suppressor in melanoma [163]. In hepatocellular carcinoma and non-small cell lung cancer (NSCLC), elevated PRKAA2 enhances cell proliferation and tumor growth. Increased PRKAA2 activity concurrently inhibits apoptosis and ferroptosis through stimulating increased SLC7A11 and GPX4 activity to counteract enhanced lipid peroxidation [72,164]. Informatics analysis indicates that PRKAA2 expression is upregulated in HNSCC patients, suggesting that it plays a similar role in ferroptosis resistance [61,165]. Increased PRKAA2 activity counteracts the metabolic-suppressive effects of cetuximab treatment in HPC-negative HNSCC [166], suggesting dual targeting with the addition of anti-ferroptotic agents as a potential strategy to overcome cetuximab resistance in PRKAA2 high tumors.

### 3.6. Growth Factor Signaling

#### 3.6.1. EGFR

Epidermal growth factor receptor (EGFR) (Figure 3) is a transmembrane tyrosine kinase that binds epidermal growth factor and related ligands to stimulate downstream proliferation signaling through the activation of multiple kinase-based cascades [167]. EGFR is upregulated in approximately 30% of HNSCC cases corresponding with increased disease risk through genomic amplification [61,168]. Increased EGFR expression in HNSCC is associated with poor prognosis, increased cell proliferation, tumor growth, and radiotherapy resistance [168]. In nasopharyngeal carcinomas, EGFR binds and stabilizes SLC7A11 at the plasma membrane, which in turn inhibits major histocompatibility complex class I (MHC-I) presentation to promote immune invasion [169]. Enhanced SCL7A11 stability, in conjunction with elevated FTH1, confers enhanced ferroptotic resistance that can be overcome with combination treatment of cetuximab with the GPX4 inhibitor RSL3 [170]. Similarly, ferroptosis is initiated in EGFR-overexpressing HNSCC cells through blocking of the EGFR ligand epiregulin, which initiates ferroptosis through increased iron accumulation and GPX4 downregulation [171]. Combining cetuximab and RSL3 further enhances ferroptosis, indicating that incorporating ferroptosis-targeting strategies is of value in combating HNSCC, particularly in cases that have acquired cetuximab resistance [172].

#### 3.6.2. KRAS

Kirsten rat sarcoma viral oncogene homolog (KRAS) (Figure 3) is critical in mediating extracellular growth signaling [62]. A member of the RAS small GTPase superfamily, KRAS is often mutated in cancer where constitutive-active forms drive mitogen-activated protein kinase (MAPK) activation and gene transcription, while concurrently silencing tumor suppressor activity in colorectal cancer [173]. In HNSCC, elevated KRAS activation is linked to tumor development and poor prognosis [62]. Similar to TP53, KRAS transcription levels can be reduced by anti-cancer drugs, including estradiol and methotrexate, offering a method to blunt KRAS signaling in some tumors [62]. KRAS acts as a ferroptotic biomarker in pancreatic cancer, where it regulates ferroptosis resistance through lipid biosynthesis and antioxidant responses [62,174]. Mutationally activated KRAS signaling also suppresses pancreatic tumor immunity by contributing to damage-associated molecular patterns released by ferroptotic cells that are subsequently phagocytosed by macrophages in the tumor microenvironment in response to an iron-rich diet [175]. Such tumors with high macrophage iron content correlate with poor survival [29]. In HNSCC, KRAS is a hub gene that drives ferroptosis, with elevated protein and mRNA expression levels observed in HNSCC patients [62]. Higher KRAS levels are also associated with changes in the tumor immune microenvironment, causing greater infiltration of CD8+ and CD4+ T cells, and increased M1 macrophage polarization through the STAT3 pathway activation [30,62]. In some instances, mutated KRAS can also promote SLC7A11 activity through ATF4 to drive extrinsic ferroptosis resistance [114]. The recent advances in drugging KRAS in pancreatic cancer could provide therapeutic opportunity for ferroptosis targeting in HNSCC and other cancers where activated KRAS or cases with elevated expression is inhibitory [176].

#### 3.6.3. HRAS

Harvey rat sarcoma virus (HRAS) (Figure 3) is another Ras-family small GTPase that is frequently is dysregulated in HNSCC [62]. Up to six percent of HNSCC patients have increased HRAS expression, with another three–four percent having mutational activation linked with oncogenic onset, aggressive disease and poor prognosis [62,177]. HRAS is a ferroptosis driver in HNSCC, where it is directly linked in ferroptosis through the modulation of GPX4 activity leading to increased antioxidant and reduced lipid peroxidation product levels [62]. Elevated HRAS is associated with changes in the tumor immune microenvironment, including increased macrophage infiltration, increased CD8+ and CD4+ T cells, increased chemokines and cytokines, and high PD-L1 expression [62,178].

#### 3.6.4. PTEN

Phosphatase and TENsin homolog (PTEN) (Figure 3) is a phosphatidylinositol-3,4,5-triphospate 3-phosphatase that converts phosphatidylinositol-3,4,5-triphosphate (PIP3) to phosphatidylinositol 4,5-bisphosphate (PIP2) [179]. PTEN functions as a tumor suppressor by keeping the levels of PIP3 produced by phosphorylation of PIP2 due to phosphoinositide 3-kinase (PI3K) activity in check [180]. Loss of PTEN function occurs in approximately 30% of HNSCC, resulting in increased activation of PI3K and downstream AKT/mTOR signaling [181,182]. HNSCC tumors with PTEN loss of function display increased tumor growth, apoptosis resistance and poor overall survival [62]. Informatic and transcriptomic analysis of HNSCC tumors and cell lines identified PTEN as a ferroptosis hub gene, serving as a negative regulator of ferroptosis where PTEN loss in HNSCC leads to increased ferroptosis resistance [62,183]. Similar effects on ferroptosis have been reported in pancreatic and lung cancer [62]. PTEN expression levels are decreased upon treatment with the common chemotherapeutics cisplatin and fluorouracil (5-FU), leading to increased ferroptosis resistance [62]. Tumors with low PTEN expression are associated with an immune suppressive microenvironment, further compounding combination strategies that target ferroptosis with ICIs [184].

#### 3.6.5. AURKA

Aurora kinase A (AURKA) (Figure 4) is a cytoplasmic serine/threonine kinase essential for spindle assembly during cell division [185]. Increased AURKA expression and activity is found in many cancer types, resulting in centrosome amplification leading to aneuploidy and reduced cell cycle checkpoint sensitivity [186]. Increased AURKA activity impacts several oncogenic pathways, including PI3K/Akt, mTOR and beta-catenin/Wnt-based signaling [59]. In HNSCC, AUKRA is associated with increased tumor risk, invasion, poor prognosis and cisplatin resistance [59,61]. In accordance with driving aggressive disease, AURKA is highly expressed in HPV-negative HNSCC patients harboring TP53 mutations [59,168]. High AURKA expression confers ferroptosis resistance in multiple carcinoma types, including HNSCC, where it is part of a ferroptotic prognostic gene signature in TP53 mutant HPV-negative patients [187]. Knockdown of overexpressed AURKA in esophageal squamous carcinoma cell lines results in decreased GPX4 and SLC7A11 expression, with concurrent increased cytoplasmic iron and decreased GSH levels [188]. Further mechanistic insight into how AURKA convers ferroptosis resistance has been elucidated in lung adenocarcinoma, where AURKA knockdown induces ferroptosis through decreasing kelch-like ECH-associated protein 1 (KEAP1) expression, which in turn stimulates accumulation of NRF2 and heme oxygenase 1 (HO-1) antioxidant activity [189]. Targeting of AURKA activity with the small molecule inhibitor alisertib leads to suppression of the NRF2 and GPX4 pathways, indicating a potential means to trigger ferroptosis in tumors with high AURKA activity [190].

#### 3.6.6. FAT1

FAT atypical cadherin 1 (FAT1) (Figure 3 and Figure 4) is transmembrane member of the protocadherin family essential for intracellular adhesion [191]. FAT1 is a tumor suppressor that is frequently mutated in HNSCC and other cancer types, where mutations in the cytoplasmic region result in epithelial overgrowth that utilizes the Hippo TAZ/YAP, Wnt/beta-catenin and MAPK pathway signaling pathways [75,191]. The FAT1 cytoplasmic domain also governs actin cytoskeletal regulation at cell–cell adhesions through regulation of Ena/Vasp-mediated actin filament elongation and F-actin capping to maintain filament stability [75,191]. FAT1 can function as either a tumor suppressor or oncogene depending on the mutational context and/or tumor type [75,191]. FAT1 is one of the most frequently mutated genes in HNSCC, occurring in 23–29% of patients [61,74,75,191]. HNSCC patients with FAT1 loss of function mutations have decreased overall survival, increased lymph node metastasis, tumor recurrence and positive lymphatic vascular intravasation [75,191]. High FAT1 expression HNSCC causes increased cisplatin resistance and elevated GSH levels, indicative of protection against ROS and ferroptosis. Ferroptosis in this scenario can be resensitized through targeting of the LRP receptor and downstream Wnt signaling to decrease glutathione synthetase (GSS) activity [74]. Increased oxidative conditions due to low GSH, along with deregulation of the LRP/Wnt signaling pathway, points to FAT1 being a potential ferroptosis biomarker [74]. GSH is at the core of the GPX4-cystine/glutamate ferroptotic pathway, as it is responsible for iron homeostasis, redox balance, and lipid peroxidation [120]. As such, GSH depletion has been proposed as a treatment for cancers resistant to conventional therapies by inducing oxidative stress, where FAT1 targeting would give a congruent approach [120]. In support of this, dysregulation of glutamine metabolism, which impacts GSH levels, sensitizes FAT1-mutant HNSCC cells to ferroptosis [192].

#### 3.6.7. PAR1

Protease-activated receptor 1 (PAR1) (Figure 4), is a G-protein coupled receptor that contributes to regulating the thrombin signaling pathway in epithelium, neurons and immune cells [193]. PAR1 is activated by binding to thrombin and matrix metalloproteinase (MMP) 1, which triggers activation of the PIK3-Akt, MAPK and Rho family GTPase pathways to drive the progression of several cancer types [76,193]. PAR1 expression is increased in metastatic HNSCC cells, where HPV-negative HNSCC lines and xenografts display increased signaling utilizing the JAK2/STAT3 pathway to drive proliferation, cell survival, tumor cell migration, invasion, metastasis and EMT [76]. In HPV-negative HNSCC, PAR1 overexpression regulates of expression of the ferroptosis regulators SLC7A11, GPX4 and FTH1 signaling [76]. PAR1 inhibition by the small molecule inhibitor vorapaxar enhances erastin-mediated ferroptosis in HNSCC, identifying a rational co-target for anti-ferroptotic targeting [76].

### 3.7. Immune Signaling

#### 3.7.1. IFNG

Interferon gamma (IFNG or IFNγ) (Figure 4) is a cytokine involved in the tumor immune response, leading to loss of mitochondrial membrane potential, increased presence of ROS, JAK/STAT1 pathway activation, endoplasmic reticulum stress and apoptosis [78]. In HNSCC, IFNG has been shown to have anti-tumor properties such as anti-immune cell activation, cell cycle arrest, and apoptosis induction in advanced disease [78]. IFNG-induced apoptosis in HNSCC is triggered by the suppression of indoleamine 2.3-dioxygenase (IDO), which is a common enzyme target in many immune therapies [78,194]. While labeled as apoptosis, this manner of cell death includes several ferroptotic hallmarks such as accumulation of ROS, induction of oxidative-stress pathways and mitochondrial dysregulation. Additionally, IDO is regulated by the JAK-STAT pathway downstream of INFG stimulation, a pathway utilized in ferroptosis [78]. This is supported by observations that IFNG released from CD8+ T cells inhibits SLC7A11 in cancer cells through the STAT1 pathway to promote tumor cell ferroptosis [30]. In adrenal cell carcinoma (ACC), IFNG stimulation of the JAK/STAT pathway enhances erastin-induced ferroptosis by suppression of SLC7A11, resulting in increased lipid peroxidation and subsequent mitochondrial damage [195]. A role in HNSCC can be inferred from these studies since HNSCC ferroptosis utilizes similar JAK/STAT signaling and displays more positive outcomes in response when IFNG is activated in immune treatment [77,196].

#### 3.7.2. SOCS1

Suppressor of cytokine signaling 1 (SOCS1) (Figure 2) is a negative regulator of cytokine signaling. SOCS1 expression is induced by pro-inflammatory cytokines to generate a negative feedback loop that attenuates JAK/STAT signaling that regulates HNSCC cell growth and proliferation [44,56,91]. SOCS1 functions as a tumor suppressor by promoting protein ubiquitination, enhancing TP53 tumor suppressor functions and impairing oncogenic transcription factor expression [56,197]. Elevated SOSC1 levels promote ferroptosis by activating TP53 to downregulate SLC7A11 expression [198]. Accordingly, higher SOSC1 expression in HNSCC corresponds with improved prognosis and outcomes [56]. SOCS1 expression in HNSCC also corresponds with increased macrophage and B-cell infiltration in the TME, generating a favorable environment for pro-inflammatory T-cell infiltration [56].

#### 3.7.3. TNFSF9

Tumor necrosis factor superfamily member 9 (TNFSF9) (Figure 2) is a costimulatory ligand for the receptor TNFRSF9 (CD137) that drives anti-tumor immunity by binding to TNFRSF9 on activated T-cells [199]. TNSFSF9 stimulation on CD8+ T cells enhances proliferative signaling, survival, memory formation, cytotoxicity and cytokine production [61,199]. In HNSCC, informatic analysis indicates that TNFSF9 expression is increased in high-risk groups as part of an immune ferroptosis prognostic signature [61]. High-risk patients have increased immunosuppressive cytokines and immune checkpoint involvement that includes TNFSF9/TNFRSF9 and PD-1/PD-L1, correlating with a tumor suppressive TME and suppressed ICI response [61]. In the context of immunity and ferroptosis in HNSCC, TNFSF9 aids in predicting clinical and treatment outcomes in high-risk patients with increased mutational burden and increased immune checkpoint gene expression, suggesting that increased immunotherapy responses can be induced through co-stimulating ferroptosis in HNSCC patients with high TNFSF9 expression [61]. Preliminary correlation studies with immune-related gene signatures indicate that patients with high TNFSF9 levels are predicted to respond better to ICI-based therapies, supporting a role for TNFSF9 in predicting ICI efficacy [200].

### 3.8. Endocytosis Regulation

#### 3.8.1. CAV1

Caveolin-1 (CAV1) (Figure 3) is an integral membrane protein that serves as a scaffold linking the actin cytoskeleton to induce plasma membrane invaginations (caveolae) that facilitate non-receptor-mediated endocytosis [201,202]. Cytoplasmic regions of CAV1 are involved in several kinase-based growth signaling pathways and in inhibiting nitric oxide synthase (NOS) to limit nitric oxide and ROS production [203,204]. CAV1 has context-dependent roles in many cancers [205,206], functioning to suppress tumor formation and progression in early stages through inhibiting the unfolded protein response at the endoplasmic reticulum [207]. In advanced disease, CAV1 drives tumor cell growth, metastasis and therapeutic resistance, serving as a biomarker for poor prognosis [208,209,210]. CAV1 overexpression in HNSCC results in increased resistance to cisplatin, radiation therapy and cetuximab, corresponding with poor patient outcomes [211,212,213]. Congruent with these findings, siRNA silencing of CAV1 expression in several HPV-negative cell lines enhances ferroptosis sensitivity, indicating that CAV1 overexpression is a potent ferroptosis inhibitor in HNSCC [80,212]. Interestingly, reduced CAV1 expression is associated with increased locoregional lymph node involvement [214], presenting a potential opportunity for combating advanced disease through ferroptotic induction in such cases.

#### 3.8.2. CTTN

Cortactin (CTTN) (Figure 4) is a filamentous (F)-actin-binding protein that stabilizes newly formed actin branchpoints generated by actin-related protein (Arp) 2/3 activation [215]. Cortactin serves as a substrate for serine/threonine and non-receptor tyrosine kinases, where phosphorylation typically results in enhanced Arp2/3 network formation [216]. The CTTN gene is found within the chromosome 11q13 region, an area amplified in 25–50% of all HNSCC [82]. HNSCC with increased CTTN expression have worse outcomes, with aggressive disease characterized by increased tumor grade, lymph node metastasis, recurrence, and decreased overall survival [217]. CTTN regulates exosome secretion at sites termed invadopodia, where secreted MMPs enhance degradation of the extracellular matrix to facilitate invasion [218]. Network-based approaches of renal and lung cancer cell lines identified CTTN as a ferroptosis inhibitor. Correlative mechanistic associations between CTTN in TRF1-mediated transferrin-mediated iron uptake by Arp2/3-mediated receptor-mediated endocytosis and downregulation of actin binding by CTTN acetylation were noted as potential means of ferroptosis resistance [81,219,220].

### 3.9. Scaffolding Proteins

#### 3.9.1. TTC7B

Tetratripeptide repeat domain 7B (TTC7B) (Figure 2) is a scaffolding protein that forms a complex with phosphatidylinositol 4-kinase alpha (PI4KA) and family with sequence similarity 126 member A (FAM126A, or hyccin) to localize PI4KA to the plasma membrane for generating phosphatidylinositol 4-phosphate [221]. TTC7B interacts with proteins required for proper neuron myelination, where phosphatidylinositol 4-phosphate is a key component [221]. The importance of TTC7B activity in myelination is underscored by the identification of polymorphisms identified as risk factors for ischemic stroke susceptibility [222]. In HNSCC, elevated TTC7B correlates with poor overall survival in oral and laryngeal tumor patients [83]. Informatics work indicates that TTCTB is more frequently overexpressed in HPV-negative than in HPV-positive HNSCC, offering a unique prognostic marker based on HPV-status [83]. TTC7B upregulation strongly correlates with the expression of several ferroptosis genes including ATG5, FTH1 and NCOA4, indicating that TTC7B may be an important ferroptosis regulator in HNSCC [83]. This study also indicated that TTC7B expression correlated with the particularly interesting new cysteine-histidine-rick protein- integrin-linked kinase-parvin (PINCH-ILK-PARVIN) complex that localizes to focal adhesions to promote accelerated integrin disassembly that in turn enhances HNSCC tumor invasion and metastasis [83]. TTC7B expression also correlates with suppressive immune cell infiltration into tumors, suggesting a role in generating a tumor-favoring immune TME [83].

#### 3.9.2. TRIB3

Tribbles pseudokinase 3 (TRIB3) (Figure 4) is a pseudokinase that competes for peptide binding sites with other kinases and transcription factors to regulate their function [223]. TRIB3 regulates MAPK and PI3K signaling pathways, where overexpression drives tumor growth, stemness and drug resistance [224,225]. Evaluation of human patient samples indicates that TRIB3 overexpression in HNSCC is linked to poor outcomes by driving aerobic glycolysis through the JNK/JUN signaling pathway, enhancing proliferation, migration and tumor growth in HPV-negative cells and xenografts [226]. Examination of patient tumors by immunohistochemistry, RNA sequencing and TCGA informatic analysis indicates that TRIB3 overexpression inhibits ferroptosis in HNSCC independent of HPV status, exhibiting inhibitory effects in HPV-positive and HPV-negative cell lines [73]. Additional work in HPV-negative lines indicates that TRIB3 inhibits ferroptosis through a complex with transcription factor 4 (TCF4) and beta-catenin, blocking the transcription of arachidonate lipoxygenase 3 (ALOXE3) that is required for polyunsaturated fatty acid oxidation in generating toxic ROS levels [84]. Treatment of cells with the biflavonoid TRIB3 inhibitor hesperidin enhances ferroptosis in HPV-negative cell lines and tumor xenografts [84], presenting a viable agent to target TRIB3-mediated ferroptosis in HNSCC and other solid tumors.

### 3.10. Post-Translational Modifiers

#### SENP1

SUMO-specific protease 1 (SENP1) (Figure 2) is a cysteine protease that reverses the attachment of small ubiquitin-like modifiers (SUMOs) to specific proteins that modify their function (SUMOylation) [227]. SENP1 is overexpressed in prostate, breast, hepatocellular and lung cancer, where it serves as an oncogene by deSUMOylating c-Myc and PTEN to prevent their degradation, promoting tumor cell proliferation, cell survival and metastasis [228,229,230,231]. TCGA database analysis indicates that elevated SENP1 correlates with worse outcomes in HNSCC and is associated with increased expression of multiple ferroptosis genes [86]. SENP1 overexpression enhances growth and proliferation of HPV-negative HNSCC cell lines, where cells with SENP1 knockdown display enhanced ferroptosis through dysregulation of lipid peroxidation by acyl-CoA synthetase long chain family member 4 (ASCL4). SENP1 knockdown cells also had reduced SLC7A11 and GPX4 expression, confirming a role in ferroptosis regulation [86]. Natural and synthetic SENP1 inhibitors are a current area of anti-cancer research focus, raising the potential for future application of ferroptosis induction in HNSCC [232,233].

## 4. Conclusions

As the potential therapeutic role of ferroptosis in cancer continues to be heavily pursued [40,234], identification of clear biomarkers indicative of treatment response will be critical. While many of these will be common to several cancer types, a comprehensive understanding of cancer-specific prognostic and actionable ferroptosis biomarkers will be essential to developing and implementing future rational therapeutic approaches. Here we have identified and described 30 biomarkers from the FerroDB V3 database and additional literature validation that have been shown or are inferred to mediate ferroptosis resistance or sensitivity in HPV-negative HNSCC. A summary of their clinical characteristics, including evidence type, prognostic and therapeutic implications is shown in Table 2.

### Limitations

While the biomarkers reviewed here may provide potential means of stratifying patients for improved therapeutic outcomes, future work will likely discover additional molecules involved in ferroptosis in HNSCC and other cancer types, presenting additional challenges as well as opportunities for selective targeting. Limitations to targeting ferroptosis are well known, as it is recognized that inducing ferroptosis as an anti-cancer strategy results in undesirable increases in ROS and lipid peroxidation in healthy cells [235,236]. This in turn leads to side effects that include liver and kidney damage, cachexia, death of immune cell and loss of bone marrow function [237]. Cardiac toxicity has also been observed in patients treated with doxorubicin, where iron overload, glutathione depletion and GPX4 downregulation have been documented as a result of treatment [238]. While these side effects can be combated by restoring iron homeostasis through iron chelation, such interventions would promote tumor cell survival, thus negating anti-ferroptosis treatment [235,239]. HPV-negative HNSCC displays high levels of tumor heterogeneity that compound patient treatment and varies by different head and neck subsites [240,241]. Additionally, while PCR validation of HPV combined with p16 immunohistochemistry staining is the gold standard in determining viral involvement, inconsistencies in HPV screening and methodology may misdiagnose some patients, leading to misapplication of ferroptosis or any biomarker-based therapeutic approach. These include variability in PCR thresholds, discrepancies involving anatomical subsites and continued reliance on p16 staining as the sole determinant of HPV status [242]. Furthermore, as the evaluation of ferroptosis in cells and animal models utilizes different methodologies, standardizing and integrating results from preclinical models and patients will be essential in developing clear assays for biomarker detection and measurement of effective ferroptosis targeting [243]. Integrating ferroptosis treatments with conventional neoadjuvant and adjuvant approaches presents another challenge, given the variability of some ferroptosis biomarkers to behave as inducers or suppressors depending on the specific chemo- or radiotherapy [244].

The role of ferroptosis in cancer fitness remains to be fully elucidated with understanding of how targeting these mechanisms will provide selective eradication/targeting of cancer cells compared to normal patient cells. Some support in the current treatment strategies of kinase inhibitors support targeting pathological pathways in cancer cells. Additionally, future studies using patient plasma and serum profiles will need to be initiated to validate the central thought of using ferroptosis markers in stratifying patients towards personalized medicine. Careful consideration of these factors will be critical in exploiting ferroptosis as a treatment approach in HPV-negative HNSCC and other inherently resistant cancer types.

## Figures and Tables

**Table 1:** Summary of FerrDB v.3 results and biomarker characteristics.

Biomarker	FerrDB v.3 Validated (Driver, Suppressor, Marker, Unclassified)	Pubmed ID to Start	Clinically Validated Y/N	Role in HPV-Negative HNSCC Ferroptosis (Activator or Inhibitor)	HPV Status (−) Negative, (+) Positive, (o) Either
Iron Regulation					
FTH1	D = 1 S = 8 M = 1 U = 1	[44]	Y	Inhibitor [56,57]	− [57]
TRF1	D = 2 S = 1 M = 0 U = 0	[20]	Y	Activator [57]	− [57]
ATG5	D = 3 S = 0 M = 0 U = 0	[20]	Y	Activator [58]	− [59]
NCOA4	D = 10 S = 0 M = 1 U = 0	[20]	Y	HPV- Activator [57] HPV+ Inhibitor [44]	o [44,57]
PCBP1	D = 0 S = 4 M = 0 U = 0	[60]	Y	Inhibitor [60]	− [60]
Antiporters					
SLC7A11	D = 1 S = 32 M = 1 U = 1	[20,39,44,61,62]	Y	Inhibitor [57]	− [57,63]
SLC7A5	D = 0 S = 0 M = 0 U = screened 1	[39,61]	Y	Inhibitor [64]	− [65]
Redox Reaction					
GPX4	D = 0 S = 46 M = 4 U = 0	[20,39,44,61]	Y	Inhibitor [57]	− [57]
CISD1	D = 2 S = 1 M = 1 U = 0	[20]	Y	Inhibitor [66]	o
Transcriptional Regulation					
TP53	D = 17 S = 5 M = 0 U = 0	[39,61,62]	Y	Inhibitor/Activator [62]	− [59,62]
ATF4	D = 1 S = 8 M = 0 U = 2	[39]	Y	Inhibitor [67]	− [67]
NFE2L2	D = 1 S = 74 M = 2 U = 0	[68]	Y	Inhibitor [68]	− [68,69]
TP63	D = 0 S = 2 M = 0 U = 0	[64]	Y	Inhibitor [63]	− [63]
Cellular Metabolism					
AKR1C3	D = 0 S = 3 M = 0 U = 0	[70,71]	Y	Inhibitor [71]	− [71]
PRKAA2	D = 4 S = 7 M = 0 U = 0	[61]	Y	Inhibitor [72]	− [61]
Growth Factor Signaling					
EGFR	D = 2 S = 1 M = 0 U = 0	[61]	Y	Inhibitor [73]	− [73]
KRAS	D = 1 S = 0 M = 0 U = 0	[62]	Y	Inhibitor [62]	− [62]
HRAS	D = 1 S = 0 M = 0 U = 0	[62]	Y	Activator [62]	− [62]
PTEN	D = 4 S = 0 M = 0 U = 0	[62]	Y	Inhibitor [62]	− [62]
AURKA	D = 0 S = 4 M = 0 U = predicted 1	[61]	Y	Inhibitor	− [59]
FAT1	D = 0 S = 0 M = 0 U = 0	[61]	Y	Inhibitor [74]	− [75]
PAR1	D = 1 S = 0 M = 0 U = 0	[76]	Y	Inhibitor [76]	− [76]
Immune Signaling					
IFNG	D = 8 S = 0 M = 0 U = 0	[77]	Y	Activator [58]	− [78]
SOCS1	D = 2 S = 0 M = 0 U = 0	[44,61]	Y	Activator [56]	o [56]
TNFSF9	D = 1 S = 0 M = 0 U = 0	[61]	N	Activator [61]	− [79]
Endocytosis Regulation					
CAV1	D = 2 S = 3 M = 0 U = 0	[80]	Y	Inhibitor [56]	− [80]
Cortactin	D = 0 S = 0 M = 0 U = 0	[81]	Y	Inhibitor [81]	− [82]
Scaffolding Proteins					
TTC7B	D = 0 S = 0 M = 0 U = 0	[83]	N	Activator/Inhibitor [83]	− [83]
TRIB3	D = 0 S = 3 M = 0 U = screened 1	[73,84]	Y	Inhibitor [73]	− [73,85]
Post-translational Modifiers					
SENP1	D = 0 S = 4 M = 0 U = 0	[86]	Y	Inhibitor [86]	− [86]

**Table 2:** Summary of clinical implications for each biomarker.

Biomarker	HNSCC Evidence Type (Cell Lines, Patient Tumors, Informatics, Outcomes Inferred from Other Disease)	HPV Status (−) Negative, (+) Positive, (o) Either	Overall Prognostic Effects (Poor, Favorable)	Therapeutic Implications (Druggable, ICI, Chemotherapy, Radiation)
Iron Regulation				
FTH1	Informatics, cell lines, patient tumors, informatics [56,57]	− [57]	Poor [57]	ICI, druggable, radiation [56,57]
TRF1	Cell lines, patient tumors, informatics [57]	− [57]	Poor [57]	Druggable, radiation [57]
ATG5	Informatics, cell lines [58,59]	− [59]	Poor [58,59]	ICI [58]
NCOA4	Cell lines, patient tumors, informatics [57]	− [57]	Favorable [57]	Druggable, radiation [57]
PCBP1	Informatics, cell lines, patient tumors [60]	o	Poor [60]	Druggable, chemotherapy [60]
Antiporters				
SLC7A11	Informatics, cell lines, patient tumors, informatics [57,61]	− [57,63]	Favorable [57]	Druggable, radiation [57,61]
SLC7A5	Cell lines, patient tumors, Informatics, Inferred from other diseases [61,64,118]	− [65]	Poor [118]	Druggable, ICI, radiation [61,64,118]
Redox Reaction				
GPX4	Cell lines, patient tumors, informatics [57]	−	Poor	Druggable, radiation
CISD1	Informatics [131]	o	Poor	Druggable
Transcriptional Regulation				
TP53	Informatics [61,62], cell lines	− [62]	Poor	Druggable [61]
ATF4	Patient tumors, informatics, cell lines [67]	− [67]	Poor [67]	Druggable [67]
NFE2L2	Informatics, cell lines, patient tumors [68,69]	− [68,69]	Poor [68]	Druggable, chemotherapy [68]
TP63	Cell lines, patient tumors, informatics [64]	− [63]	Poor [64]	Druggable, ICI [64]
Cellular Metabolism				
AKR1C3	Cell lines, patient tumors, informatics [71]	− [71]	Poor [71]	Druggable, ICI [71]
PRKAA2	Informatics [61,165]	− [61]	Poor [165]	Druggable [61]
Growth Factor Signaling				
EGFR	Informatics [61,73]	− [73]	Poor [61]	Druggable [61]
KRAS	Informatics, cell lines (37560204)	−− (37560204)	Poor	Druggable (35280777)
HRAS	Informatics, cell lines, clinical trials, patient tumors [62,63,177]	− [62,177,178]	Poor [178]	Druggable [63,177]
PTEN	Informatics, cell lines [62]	− [62]	Poor [62]	ICI [184]
AURKA	Informatics, cell lines [59,61]	− [59]	Poor [59]	Druggable, ICI [59]
FAT1	Informatics, patient tumors, cell lines [61,74]	− [75]	Poor [74]	Druggable, chemotherapy [61,75]
PAR1	Informatics, cell lines, patient tumors [76]	− [76]	Poor [76]	Druggable [76]
Immune Signaling				
IFNG	Informatics, cell lines [77,78]	− [78]	Favorable [78]	ICI [77,78]
SOCS1	Informatics [56,61]	o [56]	Favorable	Druggable, ICI, chemotherapy [56,61,198]
TNFSF9	Informatics [61,79]	− [79]	Favorable [61,200]	Druggable, ICI, chemotherapy [61,200]
Endocytosis Regulation				
CAV1	Informatics, patient tumors, cell lines [56,59,85]	− [80]	Poor [80,85]	Druggable, chemotherapy [85]
Cortactin	Informatics, cell lines [81]	− [82]	Poor [81]	Druggable [81]
Scaffolding Proteins				
TTC7B	Informatics [83]	− [83]	Poor [83]	Druggable [83]
TRIB3	Informatics, cell lines, patient tumors [73,85]	o [73,85]	− poor + favorable [73]	Druggable, chemotherapy [85]
Post-translational Modifiers				
SENP1	Cell lines, patient tumors, informatics [86]	o	Poor [86]	Druggable [86]

## Data Availability

No new data were created or analyzed in this study. Data sharing is not applicable to this article.
