# Correction: Parks et al. Ferroptosis Biomarkers in HPV-Negative Head and Neck Squamous Cell Carcinoma. *Cancers* 2026, *18*, 2320

**DOI:** 10.3390/cancers18162604

**Published:** 2026-08-13

**Authors:** Sarah M. Parks, Werner J. Geldenhuys, Scott A. Weed

**Affiliations:** 1 Department of Biochemistry and Molecular Medicine, School of Medicine, West Virginia University, Morgantown, WV 26506, USA; sarah.spear@hsc.wvu.edu; 2 Department of Pharmaceutical Sciences, School of Pharmacy, West Virginia University, Morgantown, WV 26506, USA; 3 Graduate Program in Cancer Cell Biology, West Virginia University, Morgantown, WV 26506, USA

**References Errors**

In the original publication [1], references 26, 54, and 130 were erroneously included and have been removed. Reference 5 was incorrect; the proper citation is Yu, M.C.; Ho, J.H.; Lai, S.H.; Henderson, B.E. Cantonese-style salted fish as a cause of nasopharyngeal carcinoma: Report of a case–control study in Hong Kong. *Cancer Res.* **1986**, *46*, 956–961. PMID:3940655. Reference 95 was incorrect; the proper citation is Saito, Y.; Soga, T. Amino acid transporters as emerging therapeutic targets in cancer. *Cancer Sci.* **2021,** *112*, 2958–2965. With this correction, the order of some references and citations in the main text has been adjusted accordingly.

The authors state that the scientific conclusions are unaffected. This correction was approved by the Academic Editor. The original publication has also been updated.
